# Foliar applied calcium chloride alleviated drought stress in pearl millet (*Pennisetum glaucum* L.) by improving growth and yield contributing traits and antioxidant activity

**DOI:** 10.1371/journal.pone.0310207

**Published:** 2024-12-23

**Authors:** Tauqeer Ahmad Yasir, Wasif Azhar, Qaisar Ali, Muhammad Usman Masood Bhutta, Muhammad Ateeq, Allah Wasaya, Mubshar Hussain, Rehana Riaz

**Affiliations:** 1 Institute of Agronomy, Bahauddin Zakariya University, Multan, Pakistan; 2 Department of Agronomy, University of Layyah, Layyah, Pakistan; 3 Department of Sustainable Land Management, The School of Agriculture, Policy and Development (SAPD), University of Reading, England, United Kingdom; 4 State Key Laboratory of Crop Stress Resistance and High-Efficiency Production and College of Agronomy, Northwest A&F University, Yangling, Shaanxi, P.R. China; 5 Department of Botany, Bahauddin Zakriya University, Multan, Pakistan; Portuguese Catholic University: Universidade Catolica Portuguesa, PORTUGAL

## Abstract

Drought-induced stress presents a substantial threat as it disrupts the normal growth of cereal crops and leads to decreased yields. The persistent occurrence of drought conditions significantly impacts the growth and development of pearl millet. This study aimed to explore how calcium chloride (CaCl_2_) regulates the growth of pearl millet when it faces a lack of water. Over two years, field experiments were conducted at the College of Agriculture, Bahauddin Zakariya University, Bahadur Sub-Campus Layyah. During the study, we exposed pearl millet to various foliar applications of CaCl_2_ (0 mg/L, 25 mg/L, 50 mg/L, and 75 mg/L) while subjecting it to two different irrigation conditions: full irrigation and drought stress during the booting stage. Results revealed that a significant reduction in the growth (plant height; PH, stem diameter; SD, fresh leaf weight; FLW, stem fresh weight; SFW, stem dry weight; SDW, root fresh weight; RFW, root dry weight; RDW, and plant dry weight; PDW), yield (panicle length; PL, grain per panicle; GPP, grain weight; GW, thousand grain weight; TGW, grain yield; GY, biological yield; BY, and harvest index; HI), and physiological attributes (membrane stability index; MSI, and soil plant analysis development; SPAD) were found under water drought stress condition, while increment in antioxidant level was observed due to low moisture contents in soil. In both years, foliar applied CaCl_2_ enhanced all the physiological, growth and yield traits as well as some of the antioxidants like superoxide dismutase (SOD), peroxidase (POD) and ascorbate peroxidase (APX). Study concluded that a concentration of 50 mg/L of CaCl_2_ is optimal for enhancing all examined attributes of pearl millet under both drought and full irrigation conditions. The results strongly advocate for the use of CaCl_2_ as the most effective treatment for the cultivation of pearl millet in arid and semi-arid regions.

## 1. Introduction

Pearl millet (*Pennisetum glaucum* L.) is one of the important cereal crops, mostly grown in the arid and semi-arid region of Asian and African continents. It is a short duration C_4_, and Cross-pollinated crop, mostly used for dual purpose (fodder and grain) [1, 2]. It belongs to the Poaceae family and in Pakistan it is commonly known as “Bajara”. With C_4_ properties, this dual-purpose crop is valuable for both fresh biomass and grain production, especially in areas with low moisture availability. Its grains are used for making human food (bread, cookies, and flour), animal and bird feed, and production of ethanol [2, 3]. Nutritionally, pearl millet is a good source of protein, energy, different kind of macro and micro nutrients [4]. The Economic Survey of Pakistan reported that in 2020–21 pearl millet was grown on an area of 0.350 million ha, producing 0.266 million tones. This marked a 30.7 percent decrease in production compared to the previous year [5]. Pakistan climatic condition is satisfactory for millet cultivation but per hectare production is still very low in the tropical areas. The main reason is acute osmotic stress in plant due to the global warming. Yield losses of most of the cereal crops are forecasted by the increasing global mean surface temperature (GMST). Gradual rise in GMST due to global warming not only triggers drought and high temperature stresses but also increases other abiotic and biotic stresses [6].

Drought stress is one of the most devastating abiotic factors which limits agricultural productivity, threatens food security and indirectly limits the production and growth of the economy worldwide [7]. Drought stress is the condition of no or less water supply, which causes less food supply to the rapidly increasing population [8]. It causes numerous changes in plant at biochemical and physiological level [9]. Which result in altering plant water status, photosynthesis, activity of antioxidant enzymes [10]. Water deficiency also affects the membrane integrity, pigment content, water relations, osmotic adjustments, photosynthetic activity, growth, and plant yield [11]. However, in limited water supply the leaf relative water content (RWC) and dry matter accumulation have been recognized as two important developments in plant productivity, growth and the water balance of the tissues [12]. In a study by [13], pearl millet was found to have less stable seed yields compared to sorghum when assessing drought resistance. Furthermore, [14] observed that post-flowering drought in pear millet resulted in a decline in seed yield. This decline was attributed to reductions in number of ears per m^2^ and seed per ear and seed weight. Recent studies highlight that millet’s seed yield diminishes under water stress due to reductions in key yield components, as evidenced by [15]. Effect of water stress on WUE depends on plant species and phenological stage of water stress imposition and severity [16]. Reductions in seed weight can occur due to decreases in seed growth rate or the length of the seed filling period. In instances of drought stress, a significant proportion (50%) of seed yield reduction was linked to a decline in harvest index [17, 18]. Calcium (Ca) is an important nutrient for the development and growth of plants and it takes part in numerous physiological mechanisms in plants [19]. It is a crucial part of the plant cell wall and essential for new cell creation [20] and the growth of leaves and roots by triggering the numerous enzyme systems [21]. Calcium works as a multifunctional element in plants, as it is involved in many physiological mechanisms like working alongside with phytohormones, increasing the action of many crucial enzymes, maintaining cell wall structure and membrane integrity [22]. Different abiotic stresses including chilling stress, high-temperature stress, salinity, heavy metal stress and drought stress are alleviated by the calcium due to its extensive features, which make it suitable for the alleviation of these stresses [23–25]. In plants, calcium ions (Ca^2+^) occur as an authoritative secondary messenger in the signaling network pathway in plant [26]. Cytosolic Ca^2+^ is believed to be increased by numerous environmental stimuli to activate various downstream and biological responses [27] that origins the regulations in plants in injurious environmental situations [28, 29] by a decrease of the membrane lipid peroxidation (LPO) and management of antioxidant defense system that help in the survival of the plant in stressful conditions [30]. Additionally, calcium involved in signaling antidrought responses and acting as a regulator of cell metabolism [28, 31]. It is testified that foliar application of Ca^2+^ improves the tolerance of plants against drought conditions [32] and also adjusts the nitrogen assimilation, photosynthetic efficiency, stress persuaded reactive oxygen metabolism and growth performance [27].

The objective of this study was to investigate the impact of moisture stress on pearl millet and to evaluate the effectiveness of foliar application of calcium chloride (CaCl_2_) as a strategy against drought stress. Furthermore, this study assessed the efficacy of CaCl_2_ foliar application in mitigating drought stress, and determine the optimal level of CaCl_2_ for improving the growth, yield, and antioxidant activity of pearl millet under drought conditions.

## 2. Material and methods

### 2.1. Site description

**Fig 1:**
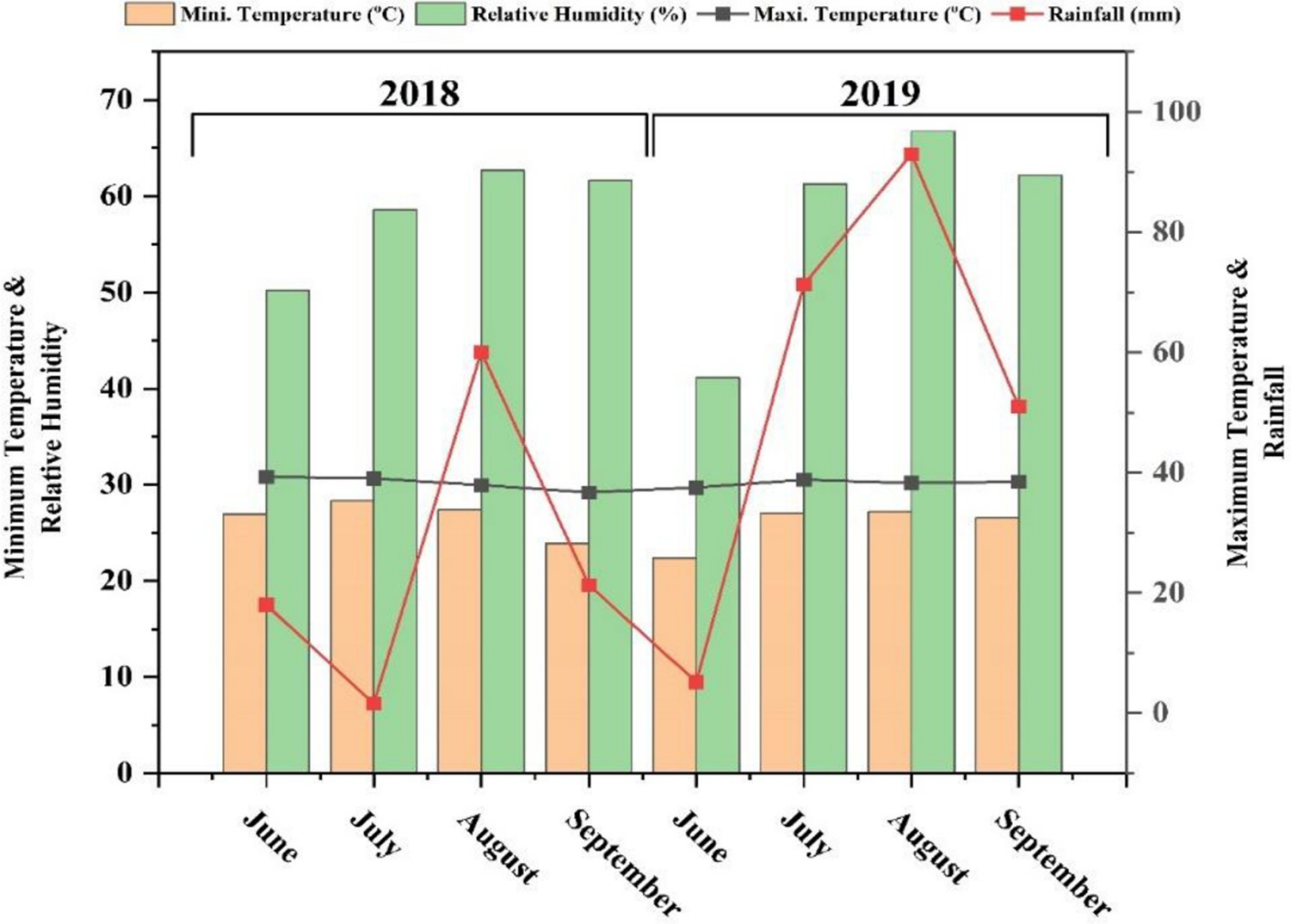
Weather data of experimental site during both years (June to September-2018 and 19).

This two-year field study was conducted at the Agronomic Research Area, BZU, Bahadur Sub Campus Layyah (longitude 70°56′38″ E, latitude 30°57′55″ N, and altitude 143 m above sea level). This area is considered an arid zone, due to the receiving annual rain fall of <1%. The soil of the experimental site has a sandy-loam soil nature with organic matter 0.35%, pH 8.3–8.4, available phosphorus 9 mg kg^-1^, total nitrogen 0.13–0.17 mg kg^-1^, available potassium 84 mg kg^-1^, electrical conductivity 0.83–1.14 dS/m, and maximum water holding capacity 28.7%. The weather data at the experimental site during both years (2018 and 2019) is given in Fig 1.

### 2.2. Experimental design and treatments

The experiments were structured using a randomized complete block design (RCBD) with a split plot arrangement having three replicates. The main plot was allocated to irrigation levels, while the sub-plots were dedicated to the application of foliar calcium chloride (CaCl_2_). Within the scope of irrigation conditions, two distinct levels were considered. The first involved regular watering, maintaining the field capacity at 100%, starting from the early stages of crop growth until reaching physiological maturity. In contrast, the second condition entailed reduced watering, specifically during critical growth stages from booting (Feekes 10.0) to heading (Feekes 10.5), with the field capacity set at 50%. Field capacity was determined through soil moisture analysis, involving weekly soil sample collection from depths of 15 and 30 cm, following the methodology of [33]. The saturation percentage (SP) of the soil was calculated, and at SP, soil moisture content was found to be 34.42%. Half of this value was designated as 100% field capacity, while half of the 100% field capacity represented the 50% field capacity. To maintain these field capacity levels, a specified quantity of water was applied whenever the soil moisture fell below the required levels following the treatment protocols. A cut-throat flume was employed to precisely dispense the necessary amount of water for each irrigation treatment, as described by [34]. In the sub-plots, calcium chloride was applied as a drought mitigating agent in three different foliar concentrations: C_0_ (Control), C_1_ (25.0 mg/L), C_2_ (50.0 mg/L), and C_3_ (75.0 mg/L). These doses were finalized on the basis of previous research.

### 2.3. Crop husbandry

For this study, the Pearl millet variety *YBS-98* was selected, and the seeds were sourced from the Ayyub Agriculture Research Institute (AARI) in Pakistan. Only healthy and uniformly sized seeds were used for sowing. At appropriate soil moisture content, the field was prepared by 2 to 3 rounds of ploughing using a common tractor-mounted plough. Thereafter, using a single row hand drill, the seeds were sown on June 7 and June 10 in the growing season 2018 and 2019 respectively.

A seeding rate of 15 kg/ha was employed with rows spaced 45 cm apart, and subsequent thinning was performed to maintain a plant-to-plant distance of 15 cm. A net plot size of 3 m × 2.7 m was used for each experimental unit. Fertilizers were applied in accordance with recommended doses, which included nitrogen (N) at 90 kg/ha, phosphorus (P) at 45 kg/ha, and potassium (K) at 0 kg/ha. Di-ammonium phosphate (DAP) was applied as a basal application at the time of sowing, while urea fertilizer was split into two applications at the first and third irrigation. Weed control was achieved through manual hoeing. All other agronomic practices were consistently carried out throughout the crop growth period. The crop was manually harvested at maturity.

### 2.4. Data collection

#### 2.4.1. Growth-related traits

*2*.*4*.*1*.*1*. Five plants were randomly selected and carefully uprooted from each sub-plot. Plant height (in centimeters) was measured using a measuring tape, extending from the soil surface to the top of the plant. Stem diameter was measured with a digital Vernier caliper at three different points on the stem and the measurements were averaged. Subsequently, leaves, stem, and roots of the selected plants were separated using a sharp knife. To measure dry weight, these plant parts were oven-dried at 70°C until a constant weight was achieved, and the weights were recorded in grams.

*2*.*4*.*1*.*2*. *SPAD value*. The SPAD value of the ten selected plants was recorded using a SPAD-502 chlorophyll meter. Taken from the flag leaf after one week of foliar application of calcium.

*2*.*4*.*1*.*3*. *Membrane stability index (%)*. The Membrane Stability Index (MSI) was determined by analyzing the electrical conductivity of leaves using purified distilled water, following the method described by [35]. This analysis was conducted at temperatures of 40°C and 100°C. Mature leaf discs weighing 0.5 grams each were placed in test tubes containing 10 ml of d_2_H_2_O in two sets. The first set was maintained at 40°C for half an hour, while the second set was kept at 100°C for 15 minutes. Subsequently, the corresponding electrical conductivity values, EC_1_ and EC_2_, were measured using a digital conductivity meter (Adawa-260, Germany).


$$Membrane\;Stability\;Index\;(MSI)=1-\frac{EC1}{EC2}\times 100$$

#### 2.4.2. Determination of yield related parameters

Panicle length (cm) was measured using measuring tape from selected plants from each experimental treatment unit. No. of grains per panicle were counted by seed counter, whereas grain weight/panicle (g), thousand grain weight (g), were calculated by electronic weighing balance from selected plants in 1 m^2^. Grain yield (kg/ha) was measured by manual harvesting and threshing of plants at maturity from selected plants in 1 m^2^ and then their seed weight was calculated using digital weighing balance. Biological yield (kg/ha) was calculated by taking the whole plant fresh and dry weight after Harvesting (Feeks Scale stage 11) and sun drying. Harvest-index was estimated by the following formula;

$$Harvest\;Index=\frac{Grain\;Yield}{Biological\;Yield}\times 100$$

#### 2.4.3. Determination of MDA and H_2_O_2_

*2*.*4*.*3*.*1*. *MDA (μM/g fw)*. The procedure of [36] was followed for measuring the malondialdehyde (MDA) content. Leaf tissue was homogenized in 10 ml of 0.25% TBA (w/v) prepared in 10% TCA. The homogenate was heated at 95°C for 30 min and centrifuged at 10,000xg for 30 min. Absorbance of the supernatant was measured at 532 nm and 600 nm. Absorbance at 600 nm was subtracted from the absorbance at 532 nm for non-specific absorbance. The MDA concentration was estimated by using an extinction coefficient of 155 mM^−1^ cm^−1^.

*2*.*4*.*3*.*2*. *H*_*2*_*O*_*2*_ *(μM/g fw)*. Hydrogen peroxide was measured by the method of [37]. Leaf tissue was homogenized in 10 ml of 0.1% (w/v) aqueous trichloroacetic acid (TCA) and centrifuged at 10,000xg for 30 min at 4°C. The reaction mixture containing the supernatant, potassium phosphate buffer and KI reagent was incubated for 1 h in dark and subsequently, the absorbance was measured at 390 nm. The concentration of H_2_O_2_ was calculated using a standard curve of H_2_O_2_.

#### 2.4.4. Determination of antioxidants

*2*.*4*.*4*.*1*. *Catalase (u/g fw)*. Catalase activity was measured according to [38]. The decrease in absorbance was recorded at 240 nm. The enzyme activity was calculated by using the H_2_O_2_ molar extinction coefficient of 36 M^−1^ cm^−1^

*2*.*4*.*4*.*2*. *SOD activity (u/g fw)*. Superoxide dismutase (EC 1.15.1.1) activity was estimated by the method described by [39]. A reaction mixture of 3 mL contained 50 mM phosphate buffer (pH 7.8), 13 mM L^-1^ methionine, 0.075 mM nitro blue tetrazolium chloride (NBT), 0.1 mM EDTA, 1 mM riboflavin, and 50 mL enzyme extract. The reaction was initiated by adding riboflavin and irradiating the tubes under two 15 W fluorescent lamps for 15 min; the reaction was terminated when the lamps were switched off and the tubes were wrapped in black polythene bags. The absorbance of the irradiated solution at 560 nm was determined with a non-irradiated complete reaction mixture serving as blank. One unit of SOD activity was defined as the amount of enzyme, which decreases the absorbance reading to 50% in comparison with tubes without enzyme, and expressed as the unit of enzyme activity per gram of fresh leaf weight.

*2*.*4*.*4*.*3*. *Peroxidase activity (U/G Fw)*. Peroxidase activity was determined by the method of [40]. The enzyme activity was assayed by the determination of guaiacol oxidation by hydrogen peroxide. A 5-mL assay mixture comprised 100 mM potassium phosphate buffer (pH 7.0), 20 mM guaiacol, 10 mM hydrogen peroxide, and 1 mL of the enzyme extract. Changes in the absorbance of reaction solution (470 nm) were measured after 1 min with a spectrophotometer (UV-1800, Shimadzu, Japan). One enzyme activity unit was defined as the amount of enzyme that could decrease the absorbance value by 0.01 during the 1 min. The results were given as the unit of enzyme activity per gram of fresh leaf weight.

*2*.*4*.*4*.*4*. *Ascorbate peroxidase (U/G Fw)*. Ascorbate peroxidase activity was determined as described by [41]. All Enzyme activities were expressed as enzyme units per milligram of protein. One unit of APX activity was defined as the amount of enzyme required to reduce 1 μmol of H_2_O_2_ min^−1^, under assay conditions.

### 2.5. Statistical analyses

Collected data from the experiments was statistically analyzed using Fisher’s ANOVA technique on software Statistix 8.1. Analysis of variance (ANOVA) was calculated for both main and interaction effects. LSD (least significant difference) test was used to check the treatment differences at a 0.05 probability level. Bar graphs were designed in Origin Pro 9.1 software (Origin-Lab Corporation, Northampton, MA).

## 3. Results

### 3.1. The effect of drought and foliar application of Ca on growth parameters

As per physiological traits, the individual effect of drought stress and Ca application was significant in most of the growth-related treatments. Drought stress significantly (P≤0.05) reduced the growth regardless of foliar calcium supply (S1 Table in S1 File). During 1^st^ year experiment maximum Plant height (cm) in normal irrigation as well as in drought was recorded when calcium was sprayed at concentration of 50mg/L followed by 75mg/L and 25mg/L treatment. During 2^nd^ year experiment similar results were obtained showing maximum plant height well irrigated plants and drought stress at 50mg/L treatment. At the same time, minimum values were recorded when no treatment was applied in both water regimes (Table 1). Stem dry weight (g) minimum values were observed where no treatment was applied both in well irrigated and drought situations. Showing an increase of 9.81% and 21.81% in Stem dry weight in drought and well irrigated conditions.

**Table 1:** Effect of drought and foliar application of calcium on plant height (PH), stem diameter (SD) and fresh leaf weight (FLW) of Pearl millet.

		2018			2019		
	Calcium Levels	PH (cm)	SD (mm)	FLW (g)	PH (cm)	SD (mm)	FLW (g)
**Normal Irrigation**	**Control**	178.18±1.36c	18.000±0.58cd	54.177±1.28c	157.33±1.15f	19.33±0.88de	56.91±1.19cd
	**25.0 mg/L**	183.89±1.29b	20.667±0.88ab	58.517±1.28b	175.33±1.45c	21.33±0.88bc	61.48±1.27b
	**50.0 mg/L**	189.73±1.66a	21.333±0.33a	64.333±1.20a	199.00±1.52a	23.66±1.20a	67.55±1.21a
	**75.0 mg/L**	180.98±1.42bc	19.333±0.88bc	59.667±1.20b	186.00±1.20b	20.33±0.88cd	62.81±1.13b
**Drought**	**Control**	157.11±1.32e	12.667±0.88f	43.037±1.06f	137.00±1.20g	13.66±0.88g	45.83±1.17e
	**25.0 mg/L**	165.00±2.53d	14.333±0.33e	46.343±1.02e	164.67±0.88e	18.66±0.33e	56.54±0.89d
	**50.0 mg/L**	178.19±2.81bc	17.667±0.33d	52.760±0.91cd	174.33±1.20c	22.33±0.88ab	60.09±0.92bc
	**75.0 mg/L**	164.78±2.26d	16.667±0.33d	51.773±1.14d	169.67±0.88 d	16.33±0.88f	54.64±1.27d

Means with different letters differ significantly at *p* ≤ 0.05.

In the second year of the experiment, similar results were obtained shown in Table 2. Stem diameter increased when calcium was applied at the rate of 50mg/L in both well irrigated and drought condition. Maximum values (21.33) of stem diameter were recorded in well-irrigated conditions with 50 gm/L application of Ca showing an increase of 18.6% and 39.49% on drought. Maximum plant dry weight (g) was recorded when calcium was applied at the rate of 50 mg/L and a minimum was recorded in where no treatment was applied in both well irrigated and drought conditions respectively showing an increase of 36.93% in drought well irrigated and 11.25% on drought. Similar results were obtained in 2nd year experiment which values are compared and shown in (Table 3).

**Table 2:** Effect of drought and foliar application of calcium on stem fresh weight (SFW), stem dry weight (SDW) and root fresh weight (RFW) of pearl millet.

		2018			2019		
	Calcium Levels	SFW (g)	SDW (g)	RFW (g)	SFW (g)	SDW (g)	RFW (g)
**Normal Irrigation**	**Control**	128.54±4.64bc	86.767±1.39b	24.000±1.15d	129.81±3.75bc	79.36±1.38d	25.00±1.15d
	**25.0 mg/L**	135.43±3.21ab	86.767±1.27b	30.333±0.67c	135.46±2.92ab	87.76±1.27c	32.33±1.45c
	**50.0 mg/L**	142.99±1.69a	95.273±1.17a	38.000±1.53a	145.99±1.93a	96.27±1.16a	39.00±1.52a
	**75.0 mg/L**	136.70±4.42ab	88.227±0.92b	32.333±0.67b	137.70±3.99cd	91.56±1.12b	35.66±1.20b
**Drought**	**Control**	102.46±2.51e	61.803±1.42e	19.667±1.20e	116.46±3.88d	60.47±1.21g	20.66±1.20e
	**25.0 mg/L**	110.56±2.96de	67.100±1.15d	20.333±0.88e	120.23±2.57cd	67.76±0.83f	24.66±0.88d
	**50.0 mg/L**	116.28±3.54cd	75.287±1.04c	23.667±0.88d	127.28±2.96bcd	76.28±1.04d	32.66±1.20bc
	**75.0 mg/L**	111.47±3.73de	69.480±0.61d	21.000±1.00e	122.13±3.99cd	71.81±0.92e	26.33±0.88d

Means with different letters differ significantly at *p* ≤ 0.05.

**Table 3:** Effect of drought and foliar application of calcium on root dry weight (RDW) and plant dry weight (PDW) of pearl millet.

		2018		2019	
	Calcium Levels	RDW (g)	PDW (g)	RDW (g)	PDW (g)
**Normal Irrigation**	**Control**	18.000±0.58c	241.33±8.38cd	17.33±1.20cd	243.67±7.31cd
	**25.0 mg/L**	21.333±0.33b	246.67±3.33c	20.00±1.52bc	248.67±7.57c
	**50.0 mg/L**	26.333±1.20a	330.00±7.37a	27.33±1.20a	333.00±7.21a
	**75.0 mg/L**	21.333±0.88b	273.33±8.38b	22.00±1.15b	277.00±6.42b
**Drought**	**Control**	13.333±0.88e	214.00±6.76f	14.33±0.88d	216.67±8.17e
	**25.0 mg/L**	15.000±0.58de	223.33±7.75e	15.66±1.20cd	225.33±3.21e
	**50.0 mg/L**	17.333±0.88c	238.00±8.96d	20.00±1.52bc	240.00±3.33f
	**75.0 mg/L**	15.333±0.88d	224.00±4.48e	15.66±1.20cd	225.33±8.45e

Means with different letters differ significantly at *p* ≤ 0.05.

### 3.2. The effect of drought and foliar application of Ca on yield related traits

As per physiological traits, the individual effect of drought stress and Ca was significant in their interaction. Drought stress significantly (P≤0.05) reduced the yield and yield components regardless of foliar calcium supply (S2 Table in S1 File). Foliar treatment of Ca was an effective approach in improving the yield and related traits viz. Panicle Length (cm), Grain weight/panicle (g), No. of grains/panicle, thousand grain weight (g), Grain yield (kg/ha), Biological yield (kg/ha), and Harvest index (%). In 1^st^ year experiment maximum Panicle Length (cm) (23.06) in normal irrigation and (17.05) in drought was recorded at the level of 50mg/L Showing an increase of 36.69% increase in well irrigated situation and 23.19% increase in drought stress and the minimum value was recorded where only distill water was sprayed in both regimes. In 2^nd^ year experiment similar results were obtained showing a maximum panicle length with 25.88% and 29.51% increase in well irrigated and drought conditions respectively (Table 4). In 1^st^ year the maximum Number of Grains per Panicle (848) was observed in well irrigated and (753) drought condition where 50mg/L of Ca was applied while the minimum was observed where only distill water was sprayed. Showing an increase of 10.50% on well irrigated condition and 8.6% in drought stress with foliar applied calcium. In 2^nd^ year similar results were obtained with showing an increase of 10.92% in well irrigated situation and 8.96% in drought (Table 4). In 1^st^ year experiment in the case of Grain weight per plant (g) and thousand grain weight (g) maximum values were recorded in 50mg/L of Ca treatment in well irrigated and Drought showing an increase of (41,44%) (40.06%) in drought and (51.18%) (54.79%) in well irrigated conditions in both parameters. While in 2^nd^ year showing increase of (30.33%) (51.57%) in drought and (17.47%) (39.14%) in well irrigation in both parameters. In both years experiments minimum grain weight per plant and thousand grain weight values were observed in control treatments (Tables 4 and 5).

**Table 4:** Effect of drought and foliar application of calcium on panicle length (PI), grains per panicle (GPP) and grain weight (GW) of pearl millet.

		2018			2019		
	Calcium Levels	PL (cm)	GPP	GW (g)	PL (cm)	GPP	GW (g)
**Normal Irrigation**	**Control**	16.87±0.33cd	768.00±15.50d	9.30±0.63c	18.78±0.34cd	769.00±15.50de	10.53±0.40cd
	**25.0 mg/L**	19.98±0.54b	795.33±17.16c	10.24±0.63b	19.56±0.33c	796.33±17.16c	11.57±0.52b
	**50.0 mg/L**	23.06±0.51a	848.67±19.64a	14.06±0.50a	23.64±0.77a	853.00±22.53a	12.37±0.68a
	**75.0 mg/L**	20.88±0.41b	822.33±15.92b	10.95±0.43b	21.46±0.52b	830.00±16.50b	11.08±0.62bc
**Drought**	**Control**	13.84±0.38e	693.67±21.75g	6.66±0.43d	15.42±0.51f	713.67±15.63f	8.21±0.29f
	**25.0 mg/L**	15.48±0.38d	721.33±19.35f	7.52±0.34d	17.40±0.42de	752.33±16.94e	8.99±0.44e
	**50.0 mg/L**	17.05±0.44c	753.33±23.96d	9.42±0.68bc	19.97±0.77bc	777.67±12.81cd	10.70±0.48bc
	**75.0 mg/L**	16.27±0.60cd	734.33±15.91e	7.56±0.37d	16.52±0.51ef	749.00±12.66e	9.77±0.26d

Means with different letters differ significantly at *p* ≤ 0.05.

**Table 5:** Effect of drought and foliar application of calcium on thousand grain weight (TGW), grain yield (GY) and biological yield (BY) of pearl millet.

		2018			2019		
	Calcium Levels	TGW (g)	GY (kg/ha)	BY (kg/ha)	TGW (g)	GY (kg/ha)	BY (kg/ha)
**Normal Irrigation**	**Control**	7.41±0.35c	2557±172.8d	6260.3±170.9d	8.66±0.35e	2325±162.56b	6461.3±203.34d
	**25.0 mg/L**	8.66±0.39b	2672±164.5c	6893.7±172.3c	9.91±0.54cd	2673.3±106.92a	7128.0±235.00b
	**50.0 mg/L**	11.47±0.35a	2932±172.6a	7354.7±173.0a	12.05±0.25a	2936.7±112.03a	7575.7±196.21a
	**75.0 mg/L**	9.77±0.22b	2731±170.9b	6934.0±167.5b	11.35±0.38b	2765.3±143.25a	7168.3±240.84b
**Drought**	**Control**	4.36±0.71d	2218±172.0h	5036.7±166.3h	7.95±0.35de	1819.7±114.34c	6304.3±275.84e
	**25.0 mg/L**	5.41±0.26cd	2343±171.7g	5162.0±176.3g	9.32±0.33cd	2044.3±113.16bc	6363.0±196.50e
	**50.0 mg/L**	6.13±0.20c	2445±183.3e	5880.0±183.3e	12.05±0.46a	2196.3±88.41b	7094.3±201.83b
	**75.0 mg/L**	5.57±0.58c	2393±175.2f	5632.3±171.7f	10.94±0.35b	2061.3±118.62bc	6833.3±201.83c

Means with different letters differ significantly at *p* ≤ 0.05.

In 1^st^ year maximum Grain Yield (kg/ha) and Biological Yield (kg/ha) was observed when Ca was applied at the rate of 50mg/L showing significant increase of (10.23%) (16.76%) in drought and (14.66%) (17.47%) in well irrigated conditions for both parameters respectively. In 2^nd^ year experiment similar results were observed with an increase of (20.72%) (12.53%) in drought and (26.28%) (17.24%) well irrigated conditions respectively. While minimum values were observed in control treatments of both parameters each year (Table 5). In 1^st^ year experiment the maximum HI was recorded as (45.27%) by the application of 25mg/L of Ca under drought condition, while in the 2^nd^ year the highest HI was recorded as (38.87%) and (38.76%) by the application of 50 & 75 mg/L of Ca under normal irrigation condition (Table 6).

**Table 6:** Effect of drought and foliar application of calcium on harvest index (HI), membrane stability index (MSI) and soil plant analysis development (SPAD) of pearl millet.

		2018			2019		
	Calcium Levels	HI	MSI	SPAD	HI	MSI	SPAD
**Normal Irrigation**	**Control**	40.76±1.64d	48.37±0.69c	17.450±0.53cd	36.02±2.64abc	46.29±1.04de	16.01±0.46d
	**25.0 mg/L**	38.69±1.41f	53.74±0.13b	18.470±0.37bc	37.66±2.55ab	54.99±1.04b	17.88±0.32c
	**50.0 mg/L**	39.80±1.41e	56.17±1.04a	23.137±0.25a	38.87±2.34a	57.42±0.64a	24.32±0.28a
	**75.0 mg/L**	39.31±1.51ef	51.62±0.99b	19.150±0.73b	38.76±3.06a	51.54±0.65c	20.4±0.72b
**Drought**	**Control**	43.92±1.96b	39.82±0.64e	12.717±0.38g	29.1±2.87d	41.07±1.34f	14.06±0.39e
	**25.0 mg/L**	45.27±1.78a	44.69±1.15d	14.743± 0.28f	32.19±2.06abcd	45.27±0.60e	15.96±0.33d
	**50.0 mg/L**	41.47±1.82d	47.45±0.49c	16.643±0.36de	30.94±0.52bcd	49.37±0.66cd	21.19±0.26b
	**75.0 mg/L**	42.38±1.82c	42.90±0.92d	15.547±0.36ef	30.12±0.88cd	44.15±0.92e	18.19±0.25b

Means with different letters differ significantly at *p* ≤ 0.05.

### 3.3. The effect of drought and foliar application of Ca on physiological parameters

In 1^st^ year experiment SPAD value was significantly decreased (S3 Table in S1 File) in drought stress and its values were minimum (12.71) when no treatment was applied in drought and maximum values (16.64) when Ca was applied at rate of 50 mg/L. In 2^nd^ year similar results were observed (Table 6). In 1^st^ year experiment Membrane stability index (%) was significantly decreased in drought stress and its values were minimum (39.82) when no treatment was applied in drought and maximum values were (47.45) when Ca was applied at rate of 50 mg/ while in normal irrigation minimum values were (48.37) at control treatment and maximum values were (56.17) when Ca was applied at rate of 50 mg/L. In 2^nd^ year experiment similar results were obtained (Table 6).

### 3.4. The effect of drought and foliar application of Ca on antioxidant activity

**Fig 2:**
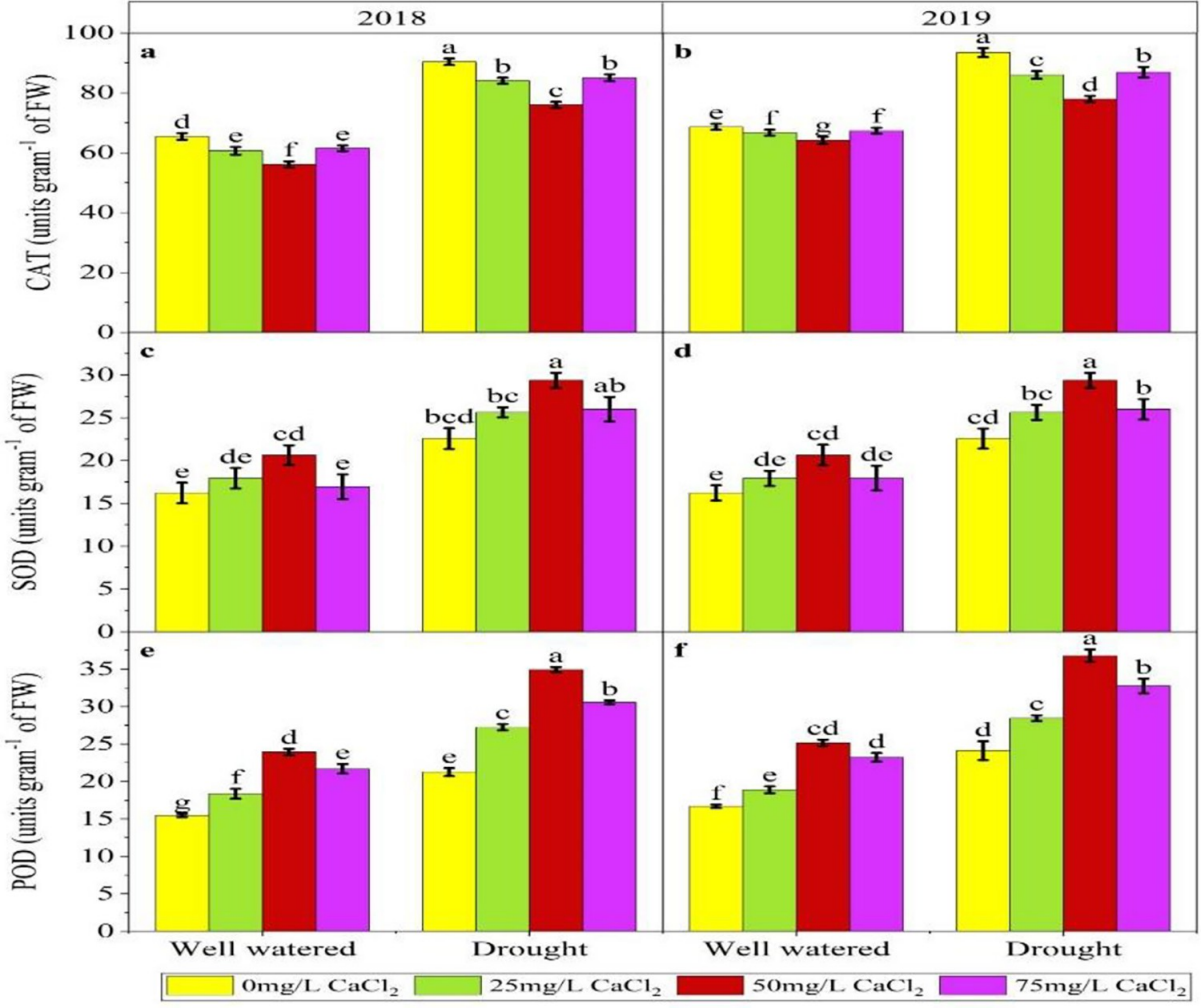
Effect of CaCl_2_ on catalase (CAT), superoxide dismutase (SOD) and peroxidase (POD) under moisture stress condition in Pearl millet.

In well irrigated situation the maximum increase in level of catalase was observed (65.44) where no treatment was applied on both years and when Ca foliar application was done at 50 gm/L the value (56.13) of catalase was decreased by 14.22% followed by the 25 and 75gm/L treatments. While in drought condition untreated plants show maximum value (90.353) of catalase and the plants treated with 50 gm/L of Ca had shown decreased value (76.003) of catalase followed by 25 and 75 gm/L showing decrease of 15.83%. In 2^nd^ year similar results were obtained with decrease of 17.20% in Drought and in normal irrigation showed decrease of 6.58%. In 1^st^ years of experiment SOD in well-watered the minimum concentration of SOD (16.21) was observed when no treatment was applied and when Ca was applied at 50 mg/L the maximum values was (20.63) followed by the 25 and 75 mg/L treatments showing increase of 27.26% while in drought minimum SOD value (22.57) was obtained in control treatment and maximum concentration (29.36) was observed when Ca was applied at rate of 50 mg/L with increase of 30.08%. While in 2^nd^ year experiment minimum concentration (16.67) of SOD was observed in control of both regimes condition and maximum was observed in 50 mg/L treatment (Fig 2). In 1^st^ year experiment Peroxidase was significantly decreased in drought stress and its values were minimum (21.25) when no treatment was applied in drought and maximum values were (34.92) at rate of 50 mg/L while in normal irrigation minimum values were (15.47) at control treatment and maximum values were (23.95) at rate of 50 mg/L. In 2^nd^ year experiment in both water regimes minimum value was recorded in control and maximum was recorded at 50 mg/L treatment (Fig 2).

### 3.5. The effect of drought and foliar application of Ca on H_2_O_2_, MDA and ascorbate levels

**Fig 3:**
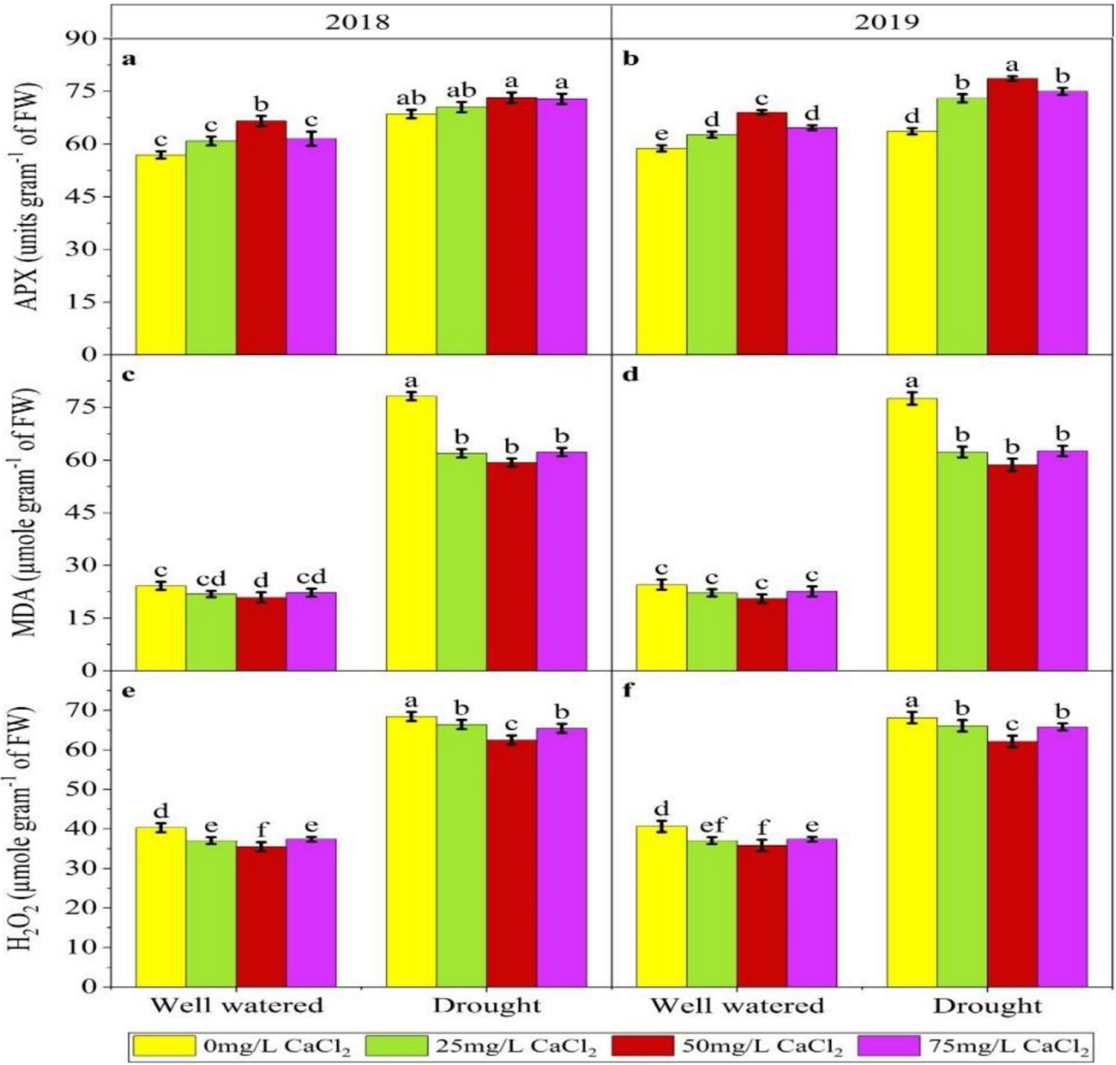
Effect of CaCl_2_ on ascorbate peroxidase (APX), monodehydroascorbat (MDA) and hydrogen per oxide (H_2_O_2_) under moisture stress condition in Pearl millet.

In 1^st^ year experiment in drought conditions maximum value (68.44) were observed in control and minimum (62.48) were observed in 50mg/L treatment showing decrease of 8.70%. In normal irrigation maximum (40.30) in control and minimum (35.46) was recorded where Ca was applied at rate of 50mg/L showing 12.01% decrease. In 2^nd^ year of experiment similar results were obtained. In drought showing a decrease of 8.75% and in case of normal irrigation maximum with decrease of 11.91% (Fig 3). MDA was significantly affected by the foliar application of Ca. In 1^st^ year experiment in drought maximum value (78.16) was recorded in control while the minimum value (59.28) was recorded in 50mg/L treatment with decrease of 24.15% while in normal irrigation maximum value (24.18) in control while minimum (20.88) in 50mg/L treatment showing 13.64% decrease in MDA concentration. Similar results were obtained in 2^nd^ year experiment in drought showing 24.36% decrease in drought while 16.15% decrease in normal irrigation (Fig 3). Foliar application of Ca Significantly affected the Ascorbate levels. In 1^st^ year experiment in drought minimum value (68.53) was recorded in control while the maximum value (73.20) was recorded in 50mg/L treatment with increase of 6.81% while in normal irrigation minimum value (56.90) in control while maximum (66.53) in 50mg/L treatment showing 16.92% increase in Ascorbate concentration. Similar results were obtained in 2^nd^ year experiment (Fig 3).

## 4. Discussion

The study revealed the optimal doze of Ca which can accelerate the growth and development of pearl millet and mitigate the effect of moisture stress. Two-year study confirmed that foliar application of Ca (50 mg/L) is an effective approach in improving millet growth and yield through maintenance of turgor, enhancing photosynthesis, pigment content, osmolytes accumulation as well as reduction in H_2_O_2_, MDA and Ascorbate content under water deficit conditions. Ca plays an important role in alleviating the damage to plants incurred under drought conditions as shown in (Tables 1–6). Similar alleviation activity by applying Ca has been reported for different plant species under different stress conditions [42]. Foliar applied Ca improved drought tolerance in maize [43], sugar beet [44], and wheat [45]. [29] also reported that foliar applied Ca in the presence of drought could increase the dry mass of leaves in the recovery phase. Increasing Ca availability may reduce drought damage by increasing membrane integrity [46]. The similar results were also found in our experiment (Table 6). The effects mentioned are due to calcium, which is a crucial component of the cell wall and plays a significant role in both cell division and cell enlargement [47]. Plant height improves with increasing concentration of Ca [48]. Therefore, it is likely to observe taller plants with the exogenous application of Ca (Table 4). Ca is also affecting the pH of the cells which stop the exit of solvents from cytoplasm and help in enhancing the shoot length [49]. This study found that applying calcium (Ca) helped plants to maintain their relative water content in both normal and water-deficient conditions. This maintenance of water content supports cell growth, which is primarily driven by turgor pressure. Our findings on the enhancement of plant height and stem diameter through the application of calcium are supported by the results of previous findings. Various Studies showed the beneficial effects of exogenously applied Ca in improving plant physiological performance. In *Zoysia japonica*, higher Chlorophyll contents were recorded with the application of Ca^2+^ in drought stress [32]. [29] highlighted the importance of calcium in maintaining cell membrane integrity, membrane permeability and growth. They found that foliar application of CaCl_2_ can increase dry matter production during the recovery stage after drought. These findings are in support of our findings regarding yield and yield related attributes of pearl millet (Table 5). [50] demonstrated that the beneficial effects of foliar-applied calcium on peanut primarily stemmed from growth and leaf expansion, and this application also facilitated the export of nonstructural carbohydrates leading to increased photochemical activity, especially during exposure to low night temperature (LNT) and subsequent warm recovery. Therefore, exogenous Ca^2+^ restored temperature-dependent photosynthesis feedback inhibition by improving sink demand in peanut under LNT stress. Ca^2+^ pre-treatment increased photosynthetic capacity by protecting PSII, activating POD and APX activities, and accumulation of compatible solute, i.e., sugars. Additionally, pre-treatment with Ca^2+^ also plays a role in regulating leaf surface temperature by controlling stomatal conductance. Dry matter increased by foliar application of Ca^2+^ even under drought stress conditions [43]. Multiple studies have demonstrated a decrease in growth related parameters under drought stress in maize [51] as well as in other crops such as *A*. *thaliana* [52], wheat [34] and rice [53] consistent with the findings presented in our results (Tables 4–6). Increased growth of tomato seedlings was also observed by [54], while investigating the impact of different concentrations of Ca (5 mM and 10mM) on germination and growth parameters.

[55] showed that foliar application of calcium chloride can improve the growth and function of cotton under abiotic stress condition. [55] demonstrated that the foliar application of calcium chloride enhances cotton growth and function under abiotic stress conditions. Our findings align with previous studies, such as [56], which have shown that foliar calcium application increases leaf area, stomatal conductance, and photosynthetic rate, leading to improved seed yield and physiological quality. In our experiment, we observed a significant increase in yield and related parameters with the application of calcium, consistent with the results of other researchers. The higher biomass in this study can be explained by the role of Ca in stabilizing organelle structure in photosynthetic machinery under drought condition [57]. Exogenous Ca was proven to mitigate the extent of degradation of photosynthesis pigment, ensuring normal photosynthesis in tobacco plant subjected to drought stress [57]. Both seed priming and foliar spray of Ca were found to enhance the performance of flag leaf and grain attributes, resulting in higher yields compared to untreated plants and those sprayed with water [57]. The application of foliar Ca proved highly effective in enhancing flag leaf gas exchange properties, chlorophyll b and carotenoids and grain pigments. These improvements significantly contributed to the growth of grains themselves, ultimately leading to higher grain yield and harvest index [58]. [59] reported that the addition of 0.08 g calcium significantly reduces stomatal closure in moderate and severe drought conditions by decreasing the ABA concentration. The mechanism allows CO_2_ to remain in and limits the oxidative damage evident from the high total chlorophyll and carotenoid content and further causes a high photosynthetic activity. The foliar application of 0.1% calcium on mung bean plants grown under abiotic stress conditions, as indicated by [60], optimized photosynthesis and nutrient uptake. This resulted in the plants maintaining optimal growth and biomass. [43] found that the foliar application of Ca at a concentration of 40 mg L^−1^ significantly enhances maize growth and productivity. Similarly, [61] noted that Ca exerts a multifaceted influence on productivity and grain quality with significant implications for maize yield particularly in environments with normal and water stress conditions. [62] found that exogenous calcium plays a crucial role in osmotic regulation, leading to increase leaf water potential, promotes leaf water maintenance, increases plant photosynthetic capacity and antioxidant capacity of leaves, and enhances the overall drought resistance of *H*. *bodinieri* seedlings.

Oxidative stress, a key component of environmental stress, is mitigated by increased SOD activity, providing protection from oxidative damage [63]. Our findings (Fig 2) indicate that the application of calcium increases levels of antioxidants such as SOD, POD, and APX. This aligns with the results of [58], who showed that calcium induces plant responses to drought by increasing SOD activity, reducing hydrogen peroxide (H₂O₂) concentration, and lowering malondialdehyde (MDA) concentration, as depicted in Figs 2 and 3. Foliar applied calcium reduced the rate of free radicals by increasing the activity of SOD as an enzymatic antioxidant. This implies that an increase of antioxidant enzymes effectively scavenges reactive oxygen species (ROS) to provide protection from cellular oxidative damage. It has also been reported that external Ca^2+^ can induce significant increases in SOD and POD activity in maize and cool season grasses seedlings [64].

## 5. Conclusion

In conclusion, the study indicates that the simulating drought conditions significantly affected the growth, productivity, and enzymatic activity of pearl millet, which experienced higher oxidative changes under moisture stress conditions, indicating increased stress on the plant. This may be mitigated by the foliar application of CaCl_2_, particularly at a concentration of 50 mg/L, which has the potential to enhance various growth and yield traits as well as certain antioxidant enzyme activities. This suggests that foliar application of CaCl_2_ may be used as a potential agronomic practice for improving pearl millet productivity in drought-prone areas.

## Supporting information

S1 File Supplementary tables file contains S1-S3 Tables. (DOCX)

S1 Graphical abstract Graphical representation of the effect of calcium chloride (CaCl_2_) on pearl millet under moisture stress. No CaCl_2_ was applied (Left). Various doses of CaCl_2_ were applied foliarly (Right). CAT, catalase; SOD, superoxide dismutase; POD, peroxidase; APX, ascorbate peroxidase; MDA, monodehydroascorbate; H_2_O_2_, hydrogen per oxide; PH, plant height; SD, stem diameter; FLW, fresh leaf weight; SFW, stem fresh weight; SDW, stem dry weight; RFW, root fresh weight; RDW, root dry weight; PDW, plant dry weight; PI, panicle length; GPP, grains per panicle; GW, grain weight; TGW, thousand grain weight; GY, grain yield; BY, biological yield; HI, harvest index; MSI, membrane stability index and SPAD, soil plant analysis development.
(DOCX)
