# Supplementary material for: Foliar applied calcium chloride alleviated drought stress in pearl millet (Pennisetum glaucum L.) by improving growth and yield contributing traits and antioxidant activity
Source: PLoS One. 2024 Dec 23;19(12):e0310207. doi: 10.1371/journal.pone.0310207 (PMC11666013; doi:10.1371/journal.pone.0310207)
Supplement: S1 File — (DOCX) [file pone.0310207.s001.docx]

**Supplementary Table 1:** ANOVA for the effect of drought and foliar Applied Calcium on Growth parameters of millet.

| **2018** | | | | | | | | | |
| --- | --- | --- | --- | --- | --- | --- | --- | --- | --- |
| **Source** | **DF** | **PH** | **SD** | **FLW** | **SFW** | **SDW** | **RFW** | **RDW** | **PDW** |
| **Irrigation (I)** | 1 | 1719.07** | 121.5** | 686.298** | 3969.37** | 2106.94** | 600** | 253.5** | 13824** |
| **Treatments (T)** | 3 | 277.86** | 17.833** | 110.124** | 202.72** | 234.77** | 83** | 38.556** | 3752.2** |
| **I×T** | 3 | 25.28** | 4.056** | 5.502** | 1.04 ns | 3.59 ns | 26.333** | 4.944** | 1486.7** |
| **Error** | 12 | 5.09 | 0.611 | 1.154 | 35.69 | 1.88 | 1.25 | 1.208 | 23.7 |
| **2019** | | | | | | | | | |
| **Irrigation (I)** | 1 | 1944** | 70.0417** | 375.409** | 1419.11** | 2318.11** | 287.042** | 165.375** | 1536** |
| **Treatments (T)** | 3 | 1719.61** | 45.7083** | 158.278** | 162.89** | 288.49** | 175.597** | 66.375** | 6822.33** |
| **I×T** | 3 | 53.22** | 5.1528** | 9.55** | 4.06 ns | 0.41 ns | 6.708 ns | 5.708 ns | 47.67** |
| **Error** | 12 | 1.08 | 0.3889 | 2.034 | 6.15 | 4.25 | 3.236 | 4.917 | 0.83 |

DF, Degree of Freedom, PH= Plant Height, SD= Stem Diameter, FLW= Flag Leaf Weight, SFW= Stem Fresh Weight, SDW= Stem Dry Weight, RFW= Root Fresh Weight, RDW= Root Dry Weight, PDW= Plant dry Weight

**Supplementary Table 2:** ANOVA for the effect of drought and foliar applied Calcium on Yield related parameters of millet.

| **2018** | | | | | | | | |
| --- | --- | --- | --- | --- | --- | --- | --- | --- |
| **Source** | **DF** | **PL** | **GPP** | **GW** | **TGW** | **GY** | **BY** | **HI** |
| **Irrigation** | 1 | 123.397** | 41251** | 67.2345** | 94.0104** | 834774** | 447.898** | 78.6988** |
| **Treatments** | 3 | 23.222** | 5335** | 15.5594** | 8.8888** | 93493** | 171.438** | 4.1946** |
| **I×T** | 3 | 2.229** | 166.6** | 1.2939** | 1.6355** | 8691** | 9.395** | 6.5355** |
| **Error** | 12 | 0.559 | 29.4 | 0.2688 | 0.3952 | 181 | 1.113 | 0.0522 |
| 2019 | | | | | | | | |
| **Irrigation** | 1 | 29.084** | 16960.2** | 23.2657** | 3.4732** | 125571** | 1133176** | 0.7315** |
| **Treatments** | 3 | 44.2561** | 6542.4** | 4.7211** | 20.1153** | 238460** | 1036811** | 2.27344 ns |
| **I×T** | 3 | 0.2124** | 100.6* | 0.5072* | 0.1468 ns | 16255 ns | 76298** | 5.34509 ns |
| **Error** | 12 | 0.2953 | 2.7 | 0.1436 | 0.08 | 124 | 78 | 0.03339 |

DF= Degree of Freedom, PL=Panicle Length, GPP= Grains per Panicle, GW= Grain Weight per Panicle TGW= Thousand Grain Weight, GY= Grain Yield, BY=Biological Yield, HI= Harvest Index

**Supplementary Table 3:** ANOVA for the effect of drought and foliar applied Calcium on Ascorbate H_2_O_2_, MDA, Antioxidants and physiological parameters of millet.

| **2018** | | | | | | | | |  |
| --- | --- | --- | --- | --- | --- | --- | --- | --- | --- |
| **Source** | **DF** | **MSI** | **LC** | **MDA** | **SOD** | **POD** | **CAT** | **APX** | **H2O2** |
| **Irrigation** | 1 | 460.338** | 129.131** | 11152.8** | 380.209** | 447.207** | 3155.17** | 578.987** | 4752.56** |
| **Treatments** | 3 | 63.5** | 24.173** | 148.2** | 32.22** | 134.268** | 142.23** | 53.374** | 29.23** |
| **I×T** | 3 | 0.068 ns | 2.676** | 79.6** | 2.201 ns | 6.882** | 6.93* | 7.761 ns | 1.38** |
| **Error** | 12 | 1.596 | 0.639 | 3.1 | 4.185 | 0.541 | 1.75 | 7.517 | 0.33 |
| 2019 | | | | | | | | |  |
| **Irrigation** | 1 | 330.858** | 31.7952** | 0.0044** | 500.507** | 0.007** | 2365.72** | 465.344** | 0.02042** |
| **Treatments** | 3 | 102.012** | 66.3956** | 0.01712** | 149.393** | 0.01718** | 45.13** | 166.511** | 0.01353** |
| **I×T** | 3 | 3.968 ns | 0.4768 ns | 0.00324** | 8.95* | 0.00075 ns | 88.54** | 10.358** | 0.0074 ns |
| **Error** | 12 | 1.385 | 0.5265 | 0.00063 | 0.514 | 0.00034 | 0.24 | 1.271 | 0.00018 |

DF= Degree of Freedom, MSI= Membrane Stability index, LC= Leaf Chlorophyll, MDA= Malondialdehyde, SOD= Superoxide dismutase, POD=Peroxidase, CAT= Catalase, APX=Ascorbate peroxidase, H2O2= Hydrogen peroxide
