# Supplementary figures and images for: Foliar applied calcium chloride alleviated drought stress in pearl millet (Pennisetum glaucum L.) by improving growth and yield contributing traits and antioxidant activity
Source: PLoS One. 2024 Dec 23;19(12):e0310207. doi: 10.1371/journal.pone.0310207 (PMC11666013; doi:10.1371/journal.pone.0310207)

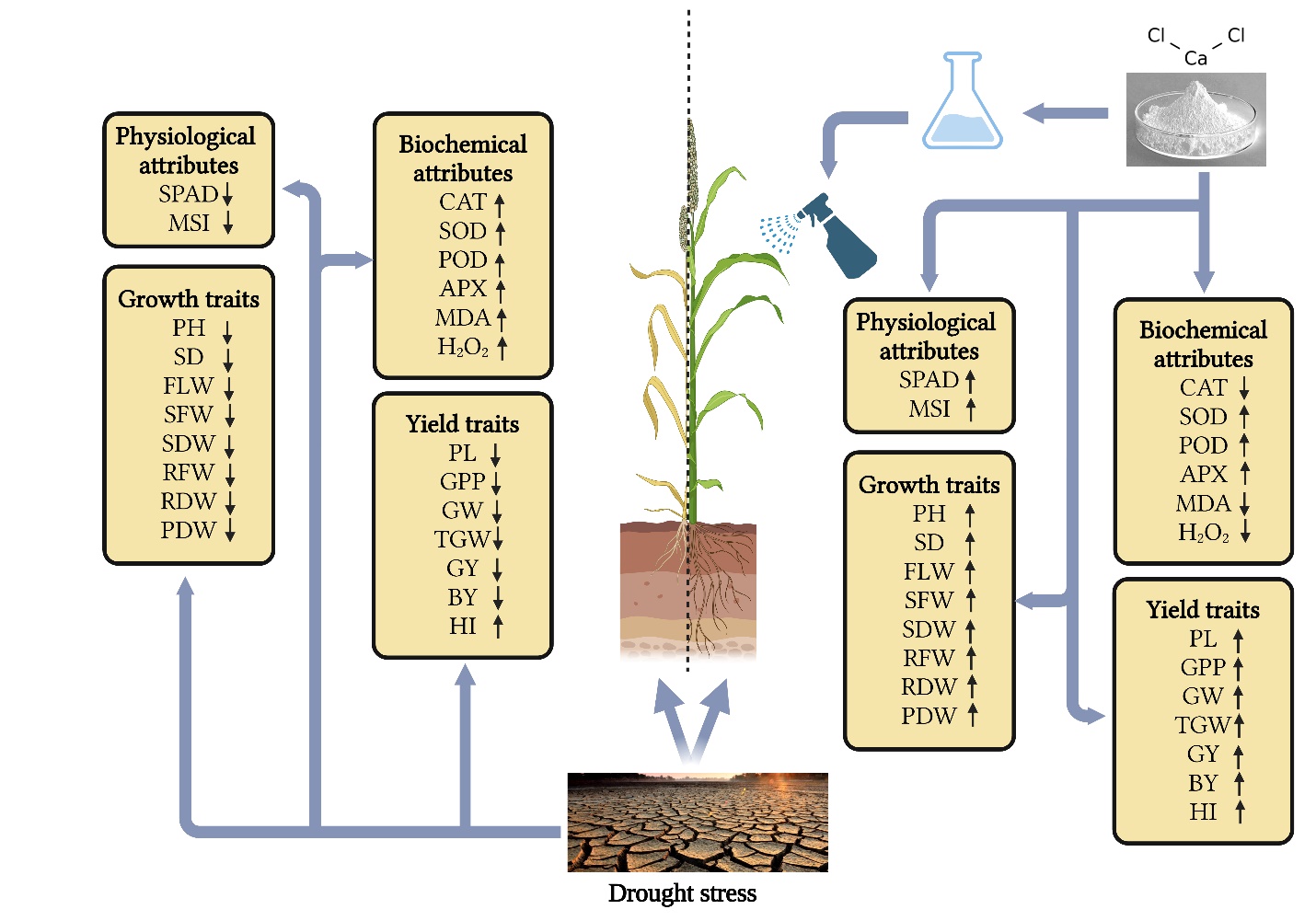

Supplement: S1 Graphical abstract — No CaCl2 was applied (Left). Various doses of CaCl2 were applied foliarly (Right). CAT, catalase; SOD, superoxide dismutase; POD, peroxidase; APX, ascorbate peroxidase; MDA, monodehydroascorbate; H2O2, hydrogen per oxide; PH, plant height; SD, stem diameter; FLW, fresh leaf weight; SFW, stem fresh weight; SDW, stem dry weight; RFW, root fresh weight; RDW, root dry weight; PDW, plant dry weight; PI, panicle length; GPP, grains per panicle; GW, grain weight; TGW, thousand grain weight; GY, grain yield; BY, biological yield; HI, harvest index; MSI, membrane stability index and SPAD, soil plant analysis development. (DOCX) [file pone.0310207.s002.docx]
